# Fermented Garlic Ameliorates Hypercholesterolemia and Inhibits Platelet Activation

**DOI:** 10.1155/2019/3030967

**Published:** 2019-12-11

**Authors:** Muhammad Irfan, Minki Kim, Kil-Soo Kim, Tae-Hwan Kim, Sung-Dae Kim, Seung-Bok Hong, Hyun Kyoung Kim, Man Hee Rhee

**Affiliations:** ^1^Department of Veterinary Medicine, College of Veterinary Medicine, Kyungpook National University, Daegu 41566, Republic of Korea; ^2^Research Center, Dongnam Institute of Radiological and Medical Sciences, Busan 46033, Republic of Korea; ^3^Department of Clinical Laboratory Science, Chungbuk Health & Science University, Cheongwon-gun, Chungbuk 28150, Republic of Korea; ^4^Department of Food Science and Engineering, Seowon University, Cheongwon-gun, Chungbuk 28674, Republic of Korea

## Abstract

Dietary cholesterol augments the lipid profile and induces the production and activation of platelets, leading to the development of atherosclerosis with detrimental effects on cardiovascular health. Ethnomedicine and Mediterranean diets are natural and cost-effective approaches against several ailments including cardiovascular diseases. In addition, fermented foods have attracted interest due to their increased nutrient profile and enhanced bioavailability and efficacy. Garlic is known to reduce cholesterol and inhibit platelet activation. Therefore, we examined whether fermented garlic could effectively ameliorate the effects of hypercholesterolemia and platelet functions in rats. Male Sprague-Dawley rats were fed a hypercholesterolemic diet and treated with spirulina and fermented and nonfermented preparations of garlic for one month. Platelet aggregation and granule secretion were assessed to evaluate platelet activation. Analysis of the liver and kidney weights and lipid and enzymatic profiles of the serum and whole blood analysis was performed. The expression levels of SREBP-2, ACAT-2, and HMG-CoA were assessed by RT-PCR, while ACAT-1 and ACAT-2 were assessed by real-time PCR, and histological changes in the liver and adipose tissues were analyzed. Both fermented and nonfermented garlic inhibited platelet aggregation and granule secretion; however, fermented garlic exhibited a greater inhibitory effect. In comparison with nonfermented garlic, fermented garlic significantly reduced liver weight and triglyceride concentrations. Fermented garlic also markedly abrogated the detrimental effects of steatosis on liver and adipose tissues. We conclude that fermented garlic significantly improved the lipid profile and modulated platelet functions, thereby inhibiting atherosclerosis- and platelet-related cardiovascular disorders.

## 1. Introduction

Cardiovascular diseases (CVDs) are among the leading causes of death in modern society [1]. One of the main underlying pathologies of CVDs is atherosclerosis, for which hyperlipidemia is a major risk factor [2, 3]. Hypercholesterolemia augments the production and hyperactivation of platelets in response to different agonists and an underlying procoagulant state, consequently leading to hypertension, atherothrombosis, myocardial infarction, and stroke [4, 5]. Activated platelets can potentially promote atherosclerosis, and the pharmacological suppression of platelets has been found to clinically reduce thrombotic events, thereby inhibiting atherosclerosis and other CVDs [5–7]. In addition to the use of clinical drugs to reduce cholesterol and platelet activation, there is a need to develop safer approaches to limit side effects and complications, which may include the use of natural products with antithrombotic properties [8]. Ethnomedicine and natural products have attracted attention for treating and preventing CVDs, and various dietary and herbal compounds have been shown to reduce atherosclerosis [9, 10]. Mediterranean diets have antiatherosclerotic and antithrombotic properties due to the presence of bioactive compounds, which can affect platelet function and reduce the risk of thrombosis development [11].

Garlic is known for its potential health benefits and is considered as one of the best disease-preventive foods as it exhibits antiplatelet, cardioprotective, procirculatory, hypolipidemic, anticancer, chemopreventive, and immune-boosting properties [12]. Considerable commercial and health benefits including an enriched flavor and improved nutrient profile may be obtained from the fermentation of food, ultimately enhancing the quality and efficacy of food or herbal products due to increased bioavailability [13, 14]. Here, we aimed to determine whether fermented and nonfermented garlic preparations could ameliorate cholesterol and modulate platelet functions in hypercholesterolemic rats.

## 2. Materials and Methods

### 2.1. Chemicals

Collagen (native collagen fibrils (type I) from equine tendons) and ADP were purchased from Chrono-Log (Havertown, PA, USA). An ATP assay kit was purchased from Biomedical Research Service Centre (Buffalo, NY, USA), and TRIZOL solution was obtained from Invitrogen (Carlsbad, CA, USA). Primers against SREBP-2, ACAT-1, ACAT-2, HMG-CoA, and GAPDH were obtained from Bioneer (Daejeon, Republic of Korea). Primer sequences have been given in Table 1. Water was obtained from J. T. Baker (Phillipsburg, NJ, USA). All chemicals were of reagent grade.

### 2.2. Preparation of Fermented and Nonfermented Garlic

Fermented garlic was prepared by the autoclaving and steaming of unpeeled garlic to produce black garlic, followed by the addition of lactic acid-producing bacteria for the second round of fermentation. Briefly, the garlic was cut in the form of beanstalk with shells and matured at 70∼75°C with 60∼70% relative humidity for 2 weeks. Here, *Bacillus subtilis* was inoculated at a weight ratio of 1% to garlic and fermented at 30∼40°C for 3 days. After fermented garlic is pulverized, it is extracted three times for 6 hours at 70∼80°C. Then, it was lyophilized and used as a sample for effect retrieval. To prepare nonfermented garlic extract, raw garlic bulbs were mashed, extracted three times with water for 6 hours at 70∼80°C, and lyophilized.

### 2.3. Animals and Dosage

Male Sprague-Dawley (SD) rats (180–200 g) were purchased from Orient Co. (Seoul, Republic of Korea) and acclimatized for one week prior to the experiment in a room with a 12/12 light/dark cycle at a temperature and humidity of 23 ± 2°C and 50 ± 10%, respectively. The rats were randomly divided into five groups (*n* = 5 in each group) as follows: normal (normal chow (purified rodent diet; AIN-76A)) (Table 2), control (hypercholesterolemic diet (HCD) (Purified diet matched to Paigen's atherogenic rodent diet; D12336) from Research diets, New Brunswick, NJ, USA) (Table 3), garlic, fermented garlic, and spirulina. Except for the normal chow group, all of the groups were fed a HCD for one week and subsequently orally administered with the vehicle, garlic (300 mg/kg), fermented garlic (300 mg/kg), or spirulina (60 mg/kg) once a day with the HCD for one month. Then, 1 h after the last oral administration, the blood and tissues were collected from the rats for analysis and further processing. Experiments were conducted following IACUC guidelines, and the protocols were approved by the Ethics Committee of the College of Veterinary Medicine, Kyungpook National University, Daegu, Republic of Korea (permit number: 2018-0090).

### 2.4. Preparation of Washed Platelets

Whole blood was collected from rats via heart puncture and transferred to a tube containing the anticoagulant acid citrate dextrose as previously described [15]. Blood was centrifuged at 170 ×*g* for 7 min to obtain platelet-rich plasma (PRP). The PRP was further centrifuged at 350 ×*g* for 7 min to isolate the washed platelets. The platelet concentration was adjusted to 3 × 10^8^ cells/mL using Tyrode's buffer (137 mM NaCl, 12 mM NaHCO_3_, 5.5 mM glucose, 2 mM KCl, and 1 mM MgCl_2_ and NaHPO_4_; pH 7.4) before using for platelet aggregation assays. All preparation procedures were performed at room temperature (23 ± 2°C).

### 2.5. *Ex Vivo* Platelet Aggregation and ATP Release Assay

Light transmission aggregometry (Chrono-Log, Havertown, PA, USA) was performed to assess platelet aggregation as previously described [16]. In brief, the washed platelets obtained from the normal, control, garlic, fermented garlic, and spirulina groups were incubated at 37°C with continuous stirring and stimulated with collagen or ADP for 5 min.

To assess the *ex vivo* effects of the given treatments on dense granule secretion, ATP assay was performed as previously described [17]. In brief, washed platelets obtained from the normal, control, garlic, fermented garlic, and spirulina groups were incubated at 37°C with continuous stirring and stimulated with collagen or ADP for 5 min. Then, the aggregation reaction was terminated, and the platelet mixture was centrifuged. The supernatant obtained was used to measure ATP secretion with a luminometer (GloMax 20/20; Promega, Madison, WI, USA) using an ATP assay kit (Biomedical Research Service Center, Buffalo, NY, USA).

### 2.6. Blood Biochemical Analysis

Fresh whole blood was taken directly from the heart of animals and transferred to EDTA tubes for plasma and hematological analyses. An automatic hematology analyzer (Sysmex XE- 2100D; Sysmex Corporation, Kobe, Japan) was used to perform a complete blood cell count on each blood sample, including hematocrit analysis. Serum samples were obtained from the blood, and total cholesterol (TC), high-density lipoprotein (HDL), low-density lipoprotein (LDL), triglyceride, GPT, GOT, and creatinine levels were analyzed using the enzymatic method (FUJI DRI-CHEM 4000i; FUJIFILM, Tokyo, Japan).

### 2.7. Histological Analysis of Liver and Adipose Tissues

The liver and adipose tissues were collected and fixed overnight in 10% formalin solution, dehydrated, embedded in paraffin, and cut into 5 *μ*m sections. Cross sections of these tissues were stained with hematoxylin and eosin (H&E), and the number of hepatocytes or adipocytes was measured using ImageJ. The results are presented as a bar graph (percent of normal tissues). For Oil Red O staining, tissues were stored at −80°C. Frozen liver tissues were sectioned with cryomicrotome and stained with Oil Red O as previously described [18].

### 2.8. RNA Extraction from Liver Tissue, Reverse Transcriptase-PCR, and Real-Time PCR

For the mRNA expression of SREBP-2, ACAT-2, and HMG-CoA, total RNA was extracted from liver tissue with TRIZOL solution using a homogenizer as previously described [19]. The RNA (2 *μ*g) was annealed with OligodT by heating at 70°C for 10 min and kept in ice for cooling for another 10 min. The reverse transcriptase reaction was carried out using a commercially available premix (Bioneer, Daejeon, Republic of Korea) by heating at 42°C for 1.5 h, and the reaction was stopped by heating at 95°C for 5 min. The resulting cDNA was added to the PCR premix (Bioneer, Daejeon, Republic of Korea) with the respective target gene primers. The PCR product was run on 1% agarose gel stained with ethidium bromide (EtBr), and the gel images were developed using an imaging system (GE Healthcare, Chicago, IL, USA). The band intensities were normalized against GAPDH, which was used as the housekeeping gene. Similarly, after reverse transcriptase, the resultant cDNA was then added with SYBR Green (Thermo Fisher, Waltham, MA, USA) with primers of GAPDH, ACAT-1, and ACAT-2 for real-time PCR analysis using CFX96 Real Time System (Biorad, Hercules, CA, USA) as previously described [19].

### 2.9. Statistical Analysis

The data were analyzed by one-way analysis of variance (ANOVA), followed by Dunnett's post hoc test to determine the statistical significance of the differences observed (SAS Institute Inc., Cary, NC, USA). All data are presented as the mean ± standard error of the mean (SEM). A *p* value of 0.05 or less was considered statistically significant.

## 3. Results

### 3.1. Effects of Fermented and Nonfermented Preparations of Garlic on Body and Organ Weights

**Figure 1:**
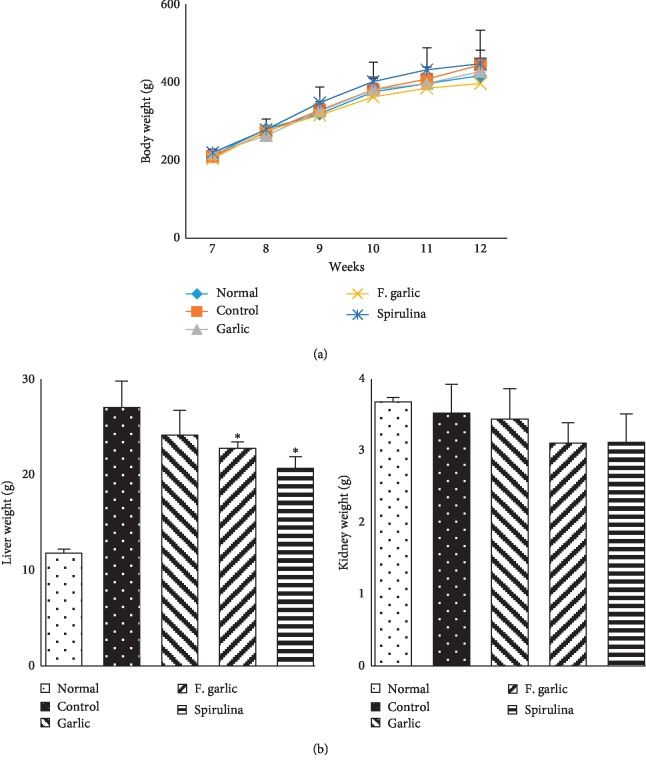
Effects of fermented and nonfermented preparations of garlic on body and organ weights. The weight of rats was examined throughout the experimental period. At the end of the experiment, the rats were killed, and the tissues were weighed. The graph represents the mean ± SD (*n* = 5). ^*∗*^*p* < 0.05 compared with the control.

There was no significant difference in the body weight among the groups. However, a high cholesterol diet significantly increased the liver weight of the control group compared with the normal chow group. A significant inhibitory effect on the liver weight was observed in the treatment groups, especially the fermented garlic and spirulina groups; however, there was no difference in the kidney weight (Figure 1).

### 3.2. Blood Analysis

As shown in Table 1, there was no significant difference in the blood cell count; however, there was a slight and insignificant decrease in hematocrit in the HCD-treated groups compared with the normal chow group. There was no significant difference compared with the control group (Table 4).

### 3.3. Fermented and Nonfermented Preparations of Garlic Inhibit Agonist-Induced Platelet Aggregation and ATP Release

**Figure 2:**
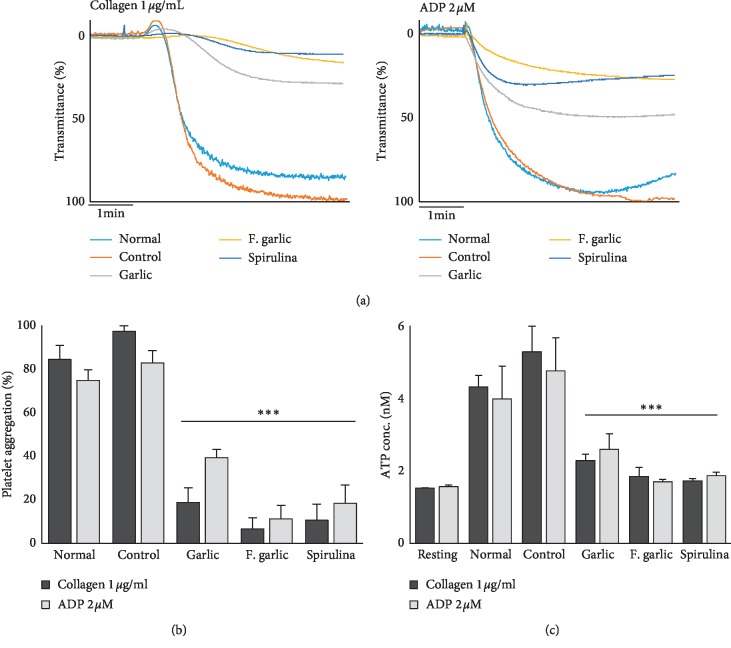
Fermented garlic inhibits agonist-stimulated platelet aggregation and granule secretion. (a, b) Washed platelets obtained from the normal chow (normal), high cholesterol diet (control), garlic, fermented garlic (F. garlic), and spirulina groups were incubated at 37°C with continuous stirring and stimulated with collagen or ADP for 5 min. (c) Washed platelets were incubated at 37°C with continuous stirring and stimulated with collagen or ADP for 5 min. Following the termination of the platelet aggregation reaction, the concentration of ATP was assessed using a luminometer. The results are presented as the mean ± SD (*n* = 5). ^*∗∗∗*^*p* < 0.001 compared with the control.

To assess the *ex vivo* effects of the treatments on platelet aggregation, washed platelets were isolated from the blood of different groups of rats, and platelet aggregation assay was performed. Collagen- and ADP-induced platelet aggregation was markedly inhibited in all the treatment groups. Interestingly, the platelets of the control group were hyperactivated compared with those of the normal chow group. Fermented garlic exhibited a greater inhibitory effect on platelet aggregation (Figures 2(a)and 2(b)).

Activated platelets release the contents of granules such as alpha and dense granules, and these enhance platelet activation including the intracellular signaling pathway. The early phases of platelet activation are characterized by the rapid release of ATP [20]. Therefore, we assessed collagen- and ADP-induced ATP secretion. As shown in Figure 2(c), ATP release from dense granules in collagen- and ADP-stimulated platelets was significantly inhibited in all the treatment groups. A greater inhibitory effect was observed with fermented and nonfermented garlic preparations. These results suggest that the given treatments could exert antiplatelet effects by suppressing platelet granule secretion.

### 3.4. Effects of Fermented and Nonfermented Preparations of Garlic on Serum Cholesterol and Enzymes

**Figure 3:**
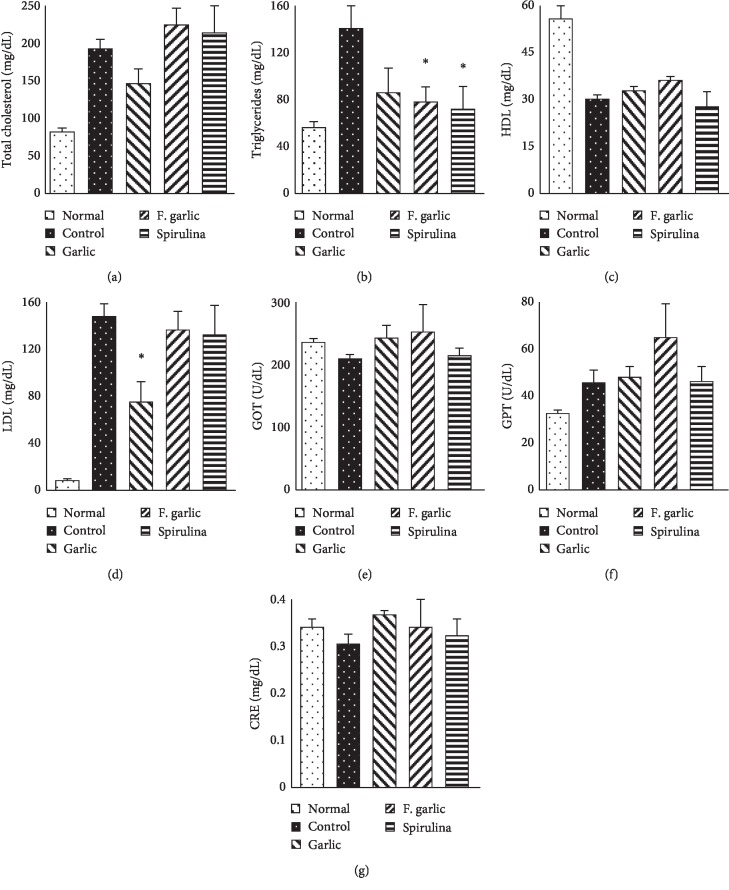
Effects of fermented and nonfermented preparations of garlic on serum cholesterol and enzymatic profile. Blood was withdrawn without anticoagulants, and the lipid profile and enzyme levels of the collected serum were analyzed. The graph represents the mean ± SD (*n* = 5). ^*∗*^*p* < 0.001 compared with the control.

TC, LDL, and triglycerides are markers of increased bad cholesterol in the body.  Figure 3 shows the inhibition of triglycerides in all the treatment groups; a significant reduction was observed in the fermented garlic and spirulina groups. LDL was greatly reduced in the garlic group; however, the effect on HDL was not significant. In addition, GPT was higher in the HCD groups than in the normal group; however, the difference was not statistically significant. Overall, there was no difference in the serum levels of liver and kidney enzymes.

### 3.5. Downregulation of SREBP-2, ACAT-2, and HMG-CoA Expression

**Figure 4:**
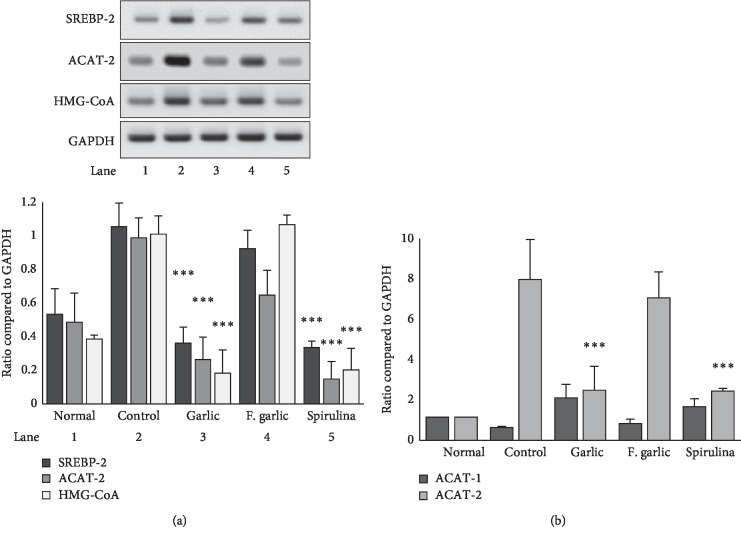
Effect of fermented and nonfermented preparations of garlic on expression of SREBP-2, ACAT-1, ACAT-2, and HMG-CoA. The liver tissue from rats treated with various samples and fed a high cholesterol diet was excised, and RNA was extracted by homogenizing with TRIzol solution. cDNA was obtained using reverse transcriptase, and the resulting cDNA was subjected to PCR with the primers of SREBP-2, ACAT-2, and HMG-CoA. GAPDH was used as the housekeeping gene. The obtained product was electrophoresed, and gel images were obtained as shown in (a). Gel electrophoresis was performed in triplicate. The intensity of gel images was quantified using ImageJ software and normalized against the housekeeping gene GAPDH as shown in bar graph (a). (b) After reverse transcriptase, the resultant cDNA was then added with SYBR Green, with primers of GAPDH, ACAT-1, and ACAT-2 for real-time PCR analysis. ^*∗∗∗*^*p* < 0.001 compared with the control.

The measurement of sterol regulatory element binding protein 2 (SREBP-2) is an important determinant of lipogenic gene transcription in the liver, and acetyl-CoA acetyltransferase 2 (ACAT-2) and *β*-hydroxy *β*-methylglutaryl-CoA (HMG-CoA) reductase are related to cholesterol and fatty acid biosynthesis [21, 22]. Spirulina is known to reduce the effects of hypercholesterolemia by regulating SREBP-2, ACAT-2, and HMG-CoA [23, 24]. Therefore, we used spirulina as a positive control. Based on our results, the mRNA expression levels of HMG-CoA, ACAT-2, and SREBP-2 in rat liver tissues fed with only a HCD were increased. On the other hand, the mRNA expression levels in the treatment groups were decreased. However, the decreased expression of HMG-CoA, ACAT-2, and SREBP was only significant in the garlic and spirulina groups (Figure 4(a)). We also measured mRNA levels of ACAT-1 and ACAT-2 in liver tissues by real-time PCR and found downregulation of ACAT-2 by the spirulina and garlic-treated groups (Figure 4(b)).

### 3.6. Fermented Garlic Prevents Steatosis

**Figure 5:**
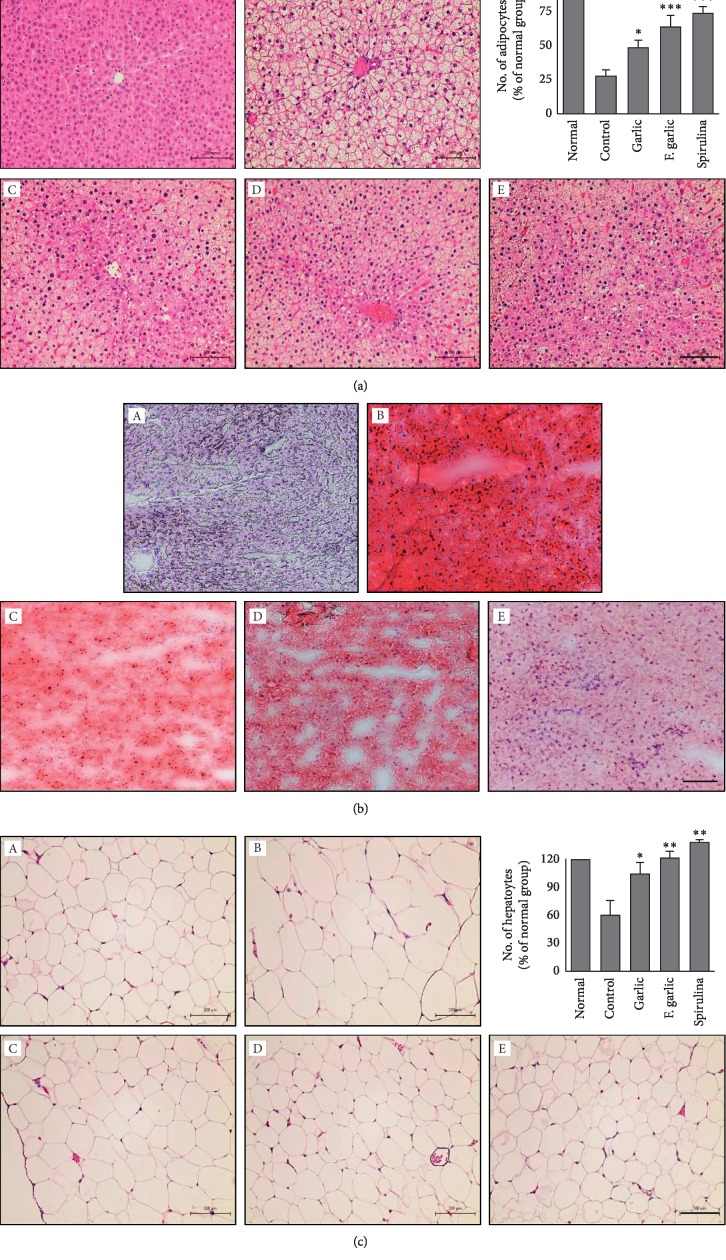
Fermented garlic prevents steatosis of liver and adipose tissues.(a‐c) Liver and adipose tissues were collected, fixed, and stained with H&E (a, c) and Oil Red O (b). (a–c) The tissues from the (A) normal, (B) control, (C) garlic, (D) fermented garlic, and (E) spirulina groups. The scale bar represents 100 *μ*m. The graph represents the mean ± SD (*n* = 3). ^*∗*^*p* < 0.05, ^*∗∗*^*p* < 0.01, and ^*∗∗∗*^*p* < 0.001 compared with the normal group.

H&E staining revealed that the histology of liver tissue in the normal group was regular and consistent with typically arranged hepatic cords (Figure 5(a) (A)). On the other hand, the fatty degeneration (steatosis) of liver tissue was observed in HCD-fed control rats due to microvesicular and macrovesicular fat deposition (Figure 5(a) (B)). However, fatty degeneration was attenuated, especially in rats fed with fermented garlic and spirulina (Figure 5(a) (C-E)).

Oil Red O staining shown extensive red staining of lipid droplets accumulated in cytoplasm of hepatocytes in the HCD-fed control group (Figure 5(b) (B)), while the lipid accumulation was reduced, especially in rats supplemented with fermented garlic and spirulina (Figure 5(b) (C-E)).

Similarly, the size of adipocytes was increased due to fatty deposits in the control group; therefore, the number of cells per field was decreased (Figure 5(c) (B)), whereas a normal histology was observed in the fermented garlic and spirulina groups (Figure 5(c) (D-E)). The bar graphs show the histological changes in the liver and adipose tissues of different groups (Figure 5(a) and 5(c)).

## 4. Discussion

The prevalence of CVDs is high due to a modern lifestyle and unhealthy eating habits in developed countries, especially Western countries. Although there are several risk factors associated with CVDs, the pathophysiological hyperactivation of platelets and cholesterol is a notable factor, contributing to atherosclerosis and stenosis of blood vessels, which can lead to ischemia or coronary heart syndrome [1, 9]. Previous studies have reported that chronic high blood cholesterol is associated with the development of atherosclerosis. Generally, dietary cholesterol induces the production of LDL by the liver and intestine, and this change in plasma lipoproteins leads to the development of atherosclerotic lesions. In the initial steps of atherogenesis, lipid peroxides injure the vessels, enhancing the adhesion and aggregation of platelets at the site of injury [25]. Due to complications and the high cost of medicines, alternative strategies are needed for the prevention of atherosclerosis, e.g., ethnomedicine or dietary therapy. Studies have shown the potential preventive and therapeutic effects of ethnomedicine and natural approaches including Mediterranean diets against CVDs [8, 11, 26].

Garlic has been used in the treatment and prevention of CVDs and can inhibit platelet aggregation [12, 27]. Fermented foods contained improved nutrient profile with enriched flavor, enhanced efficacy, and increased bioavailability [13, 14]. In the present study, we evaluated the effects of fermented and nonfermented garlic on hypercholesterolemic rats. Spirulina is known to inhibit platelet aggregation and atherosclerosis [28]. Our results showed that platelet aggregation and granule secretion were inhibited in all the treatment groups; however, fermented garlic had a greater inhibitory effect. The liver weight of rats fed a HCD only was increased due to microvesicular and macrovesicular fat deposition. On the other hand, a significant reduction in liver weight was observed among fermented garlic- and spirulina-treated rats.

Dietary cholesterol increased the lipid profile of HCD-fed rats, and their cumulative effect could lead to atherosclerosis and other cardiovascular ailments. High levels of triglyceride-rich lipoproteins or remnant cholesterol are causal risk factors of CVDs and all-cause mortality, whereas HDL cholesterol might be a noncausal marker of cardiovascular health [29]. Our results showed that triglyceride concentration was increased in HCD-fed control rats, and its elevation was inhibited in fermented garlic- and spirulina-treated rats.

SREBP may promote the transcription of genes that participate in lipid droplet formation, and ACAT-2 and HMG-CoA may be associated with cholesterol and fatty acid biosynthesis [21, 22, 30]. ACAT-1 catalyzes the reversible reaction of the formation of acetyl-coA. ACAT-2 is responsible for the secretion of cholesteryl esters. However, the expression of ACAT-1 in rat liver is originally low [31], hence showing the low expression in our real-time PCR results. ACAT-2 is the most preferred target for studies related to hypercholesterolemia and coronary heart diseases. ACAT-2 deletion causes lowering of lipoproteins inside the body [32, 33]. In our study, the expression of SREBP-2, ACAT-2, and HMG-CoA was increased in HCD-fed control rats, which indicated that cholesterol synthesis was stimulated with cholesterol accumulation in the liver, resulting in increased liver weight; however, the expression of these genes was inhibited in garlic- and spirulina-treated rats.

The ineffectiveness of fermented garlic towards a few specific parameters may be attributed to the alteration of certain compounds during fermentation, which are more effective in downregulating these genes, or there is another possible pathway involved that may need to be explored in future studies. Prolonged and continuous lipid accumulation damages the liver by increasing the size of hepatocytes, indicating steatohepatitis. Natural plant extracts have been shown to reduce the detrimental effects of steatosis on liver by reducing fat accumulation in adipocytes [18, 30]. Based on our histological results, microvesicular and macrovesicular fat deposition in the hepatocytes and adipocytes of HCD-fed rats was increased; however, treatment with fermented garlic and spirulina effectively reduced the damaging effects of steatosis.

## 5. Conclusion

Garlic and fermented garlic significantly ameliorated liver steatosis by decreasing microvesicular and macrovesicular fat deposition, triglyceride concentration, platelet aggregation, and granule secretion in hypercholesterolemic rats. Our findings suggest that fermented garlic may exert therapeutic and preventive effects against atherosclerosis- and platelet-related cardiovascular disorders.

## Figures and Tables

**Table 1:** Primer sequences.

*Gene*		Primer sequence for RT-PCR
GAPDH	Forward	5′-CACTCACGGCAAATTCAACGGCAC-3′
	Reverse	5′-GACTCCACGACATACTCAGCAC-3′
ACAT-2	Forward	5′-AGACTTGGTGCAATGGACTCGAC-3′
	Reverse	5′-CATAGGGCCCGATCCAACAG-3′
SREBP-2	Forward	5′-GGAGCCATGGATTGCACATT-3′
	Reverse	5′-AGGAAGGCTTCCAGAGAGGA-3′
HMG-CoA	Forward	5′-CTTGACGCTCTGGTGGAATG-3′
	Reverse	5′-AGTTGGAAGCACGGACATA-3′

*Gene*		Primer sequence for real-time PCR

GAPDH	Forward	5′-CACTCACGGCAAATTCAACGGCAC-3′
	Reverse	5′-GACTCCACGACATACTCAGCAC-3′
ACAT-1	Forward	5′-AAAGAAAACGGCACAGTA-3′
	Reverse	5′-CAGTGGCTTAACCTTGAG-3′
ACAT-2	Forward	5′-GAACGTGGTGGTCCATGACT-3′
	Reverse	5′-TTCAGCAGACCTCCAACCAC-3′

**Table 2:** Purified rodent diet (AIN-76A).

	gm%	kcal%

Protein	20.3	20.8
Carbohydrate	66	67.7
Fat	5	11.5
Energy (kcal/gm)	3.9	

*Ingredient*	gm	kcal

Casein	200	800
DL-Methionine	3	12
Corn starch	150	600
Sucrose	500	2000
Cellulose	50	0
Corn oil	50	450
Mineral mix	35	0
Vitamin mix	10	40
Choline bitartrate	2	0
Total	1000	3902

**Table 3:** Purified diet matched to Paigen's atherogenic rodent diet (D12336).

	gm%	kcal%

Protein	21	20
Carbohydrate	46	45
Fat	16	35
Energy (kcal/gm)	4.13	

*Ingredient*	gm	kcal

Casein	75	300
Soy protein	130	520
DL-Methionine	2	8
Corn starch	275	1100
Maltodextrin	150	600
Sucrose	30	120
Cellulose, BW200	90	0
Soybean oil	50	450
Cocoa butter	75	675
Coconut oil, 76	35	315
Mineral mix S10001	35	0
Calcium carbonate	5.5	0
Sodium chloride	8	0
Potassium citrate	10	0
Vitamin mix V10001	10	40
Choline bitartrate	2	0
Cholesterol	12.5	0
Sodium cholic acid	5	0
FD&C red dye #40	0.1	0
Total	1000.1	4128

**Table 4:** Blood analysis.

Parameters	Normal	Control	Garlic	F. garlic	Spirulina
WBC (×10³ *μ*L)	12.48 ± 1.42	14.67 ± 1.15	17.74 ± 1.39	11.56 ± 1.18	12.09 ± 1.2
Lym (×10³ *μ*L)	10.17 ± 1.16	11.34 ± 0.76	14.09 ± 1.14	8.1 ± 1.52	9.17 ± 1.05
Neu (×10³ *μ*L)	1.84 ± 0.21	2.54 ± 0.45	2.8 ± 0.26	2.94 ± 1.3	2.31 ± 0.6
Mono (×10³ *μ*L)	0.26 ± 0.03	0.38 ± 0.08	0.44 ± 0.04	0.25 ± 0.04	0.29 ± 0.04
Eos (×10³ *μ*L)	0.13 ± 0.02	0.17 ± 0.01	0.16 ± 0.01	0.12 ± 0.01	0.13 ± 0.02
Baso (×10³ *μ*L)	0.042 ± 0.01	0.044 ± 0.0	0.066 ± 0.01	0.028 ± 0.0	0.032 ± 0.01
LUC (×10³ *μ*L)	0.036 ± 0.0	0.176 ± 0.06	0.162 ± 0.04	0.104 ± 0.04	0.140 ± 0.05
PLT (×10³ *μ*L)	960.2 ± 34.39	782.6 ± 17.75	1029.8 ± 132.7	740.6 ± 56.65	729.4 ± 33.02
RBC (×10^6^ *μ*L)	8.34 ± 0.33	6.94 ± 0.19	7.2 ± 0.25	7.29 ± 0.18	7.26 ± 0.27
HGB (g/dL)	15.3 ± 0.46	12.64 ± 0.17	12.74 ± 0.45	13.02 ± 0.43	13.02 ± 0.41
HCT (%)	49.6 ± 1.96	40.98 ± 0.82	41.58 ± 1.82	42.16 ± 1.37	42.64 ± 1.48
MCV (fL)	59.44 ± 0.36	59.08 ± 0.79	57.7 ± 0.74	57.8 ± 0.87	58.84 ± 0.87
MCH (pg)	18.32 ± 0.23	18.26 ± 0.28	17.7 ± 0.27	17.8 ± 0.28	17.98 ± 0.21
MCHC (g/dL)	30.86 ± 0.35	30.92 ± 0.28	30.66 ± 0.38	30.82 ± 0.09	30.58 ± 0.15

Rat blood was collected in EDTA tubes, and the blood cell count and hematocrit were determined. The values represent the mean ± SD (*n* = 5).

## Data Availability

All the data used to support the findings of this study are included within the article.

## Abbreviations

ACAT: Acetyl-CoA acetyltransferase
HCD: Hypercholesterolemic diet
HDL: High-density lipoprotein
H&E: Hematoxylin and eosin
HMG-CoA: *β*-Hydroxy *β*-methylglutaryl-CoA
LDL: Low-density lipoprotein
RT-PCR: Reverse-transcriptase polymerase chain reaction
SREBP-2: Sterol regulatory element binding protein 2.
